# Opportunities and challenges for deep brain stimulation electrode-guided neurofeedback for symptom mitigation in neurological and psychiatric disorders

**DOI:** 10.1186/s12984-025-01701-0

**Published:** 2025-07-18

**Authors:** Oliver Bichsel, Lukas Imbach, Roger Gassert

**Affiliations:** 1 Rehabilitation Engineering Laboratory, Department of Health Sciences and Technology, ETH Zurich, Zurich, Switzerland; 2 Department of Neurosurgery, University Hospital Zurich, University of Zurich, Zurich, Switzerland; 3 Clinical Neuroscience Centre, University Hospital Zurich, University of Zurich, Zurich, Switzerland; 4 Swiss Epilepsy Center, Klinik Lengg, Switzerland; 5 The LOOP Zurich–Medical Research Center, Zurich, Switzerland

**Keywords:** Deep brain stimulation, Neurofeedback, Parkinson’s disease, Neurological disorders, Psychiatric disorders

## Abstract

Neurological and psychiatric disorders are among the leading causes of disability worldwide. With our increasing understanding of brain circuit malfunctions leading to clinical manifestations, neuromodulation techniques intervening directly at the circuit level have gained popularity as a complement to pharmacological intervention. These approaches include deep brain stimulation (DBS) and, more recently, neurofeedback. Currently, neurofeedback mainly relies on non-invasive neuroimaging but is either confined to the experimental setting or only provides nebulous cortical feedback. Interestingly, some recent DBS systems can stream electrophysiological recordings, providing a unique opportunity for neurofeedback to self-regulate ongoing brain activity at deep brain targets. Herein, we review recent studies showing rapid learning of DBS electrode-guided neurofeedback in individuals with Parkinson’s disease, with some studies supporting improved motor outcome. We provide a perspective on further applications of DBS electrode-guided neurofeedback, which encompass a wide range of disorders currently investigated with neurofeedback, focusing on other movement disorders, epilepsy, stroke and pain. The successful translation of this novel therapy approach to clinical practice still depends on technological hurdles that need to be overcome as well as larger cohorts demonstrating a meaningful benefit. As an adjunct treatment, this technique could ultimately alleviate symptoms and reduce long-term dependence on medication and DBS.

## Introduction

Neurological and psychiatric disorders are among the leading causes of burden worldwide with respect to disability-adjusted life years [1, 2]. Thus far, common treatment approaches include pharmacological interventions, rehabilitation and psychiatric therapy, which are certainly effective in some indications [3, 4]but can at times lack temporal and anatomical precision. Neuromodulatory techniques, such as deep brain stimulation (DBS) and, more recently, neurofeedback as a means to enable the endogenous self-regulation of brain activity, are opening new therapeutic possibilities for neurological and psychiatric disorders [5, 6]. The rapidly expanding scope of DBS and neurofeedback parallels our increased understanding of how brain circuit dysfunctions lead to clinical manifestations [5, 6]. They both have contributed to circuit theories of brain dysfunction by demonstrating that localised dysfunction and intervention can have profound influences on brain-wide networks [5].

Neurofeedback started in the 1950s with experiments showing that humans could self-control electroencephalographic signals in real-time [7]. Initial evidence that a monkey (*Macaca mulatta*) could be trained to increase the activity of single neurons in the precentral cortex stems from a publication in 1969[8]. Over the past decades, these findings had a profound impact on generations of brain-computer interface researchers aiming to use brain activity to control external devices [9]. Neurofeedback currently relies mainly on non-invasive functional neuroimaging such as functional magnetic resonance imaging (fMRI), functional near-infrared spectroscopy (fNIRS) [10, 11] and electroencephalography (EEG). Yet, fMRI is confined to experimental or clinical settings and the other methods are oblivious to subcortical areas (EEG mainly records pyramidal cell activity in superficial cortex [12] and suffers from poor spatial resolution in the range of several cm; whereas the mean penetration depth of an fNIRS channel with 30 mm source-detector separation is 12.5–13 mm [13, 14]). Implanted electrodes, on the other hand, could provide electrophysiological neurofeedback from anywhere in the brain with high spatial specificity and theoretically resolve single-neuron spiking activity with the option to scale to large neuronal populations, potentially opening new therapeutic avenues.

Intracranial electrodes are now routinely implanted in functional neurosurgery, most often in the context of DBS. DBS is widely used, considered safe and has become a standard of care in patients with movement disorders [5]. It involves the implantation of electrodes into specific targets within the brain, allowing for circuit-based neuromodulation through the delivery of electrical currents from an implanted stimulator. The implantation of DBS electrodes is often performed under local anaesthesia with patients fully awake to facilitate precise stimulation mapping. Under microelectrode-recording and at times awake-testing of the behavioural response upon stimulation, electrodes are advanced along a fixed trajectory at ~ 0.1–0.5 mm steps [15]. This frame-based neurosurgical procedure boasts a trajectory accuracy superior to the single-mm accuracy achieved with comparable frameless stereotactic procedures for deep seated brain regions [16]. Once implanted, DBS devices can be accessed via an external device and stimulation settings titrated to optimise clinical effects. Serious or life-threatening adverse events, such as brain haemorrhage, are rare, occurring in less than 1–2% of patients, with less serious, typically reversible events, such as wound infection and stimulation-related side effects, occurring in up to 9% of patients [17]. The standard DBS electrode configuration is quadripolar, with four 1.5 mm cylindrical electrode contacts spaced 0.5–1.5 mm apart from each other at the tip of the probe (1.27 mm in diameter), or octopolar, with the middle two contacts split up into three segments enabling directional stimulation [18]. Many other electrode configurations exist, which vary with regard to spacing between contacts as well as the number, directionality and shape of the contacts [18]. DBS then seems to act over variable time spans and through multimodal mechanisms that are not limited to the inhibition and excitation of brain circuits [19]. With future breakthroughs in the designs of batteries and electrodes as well as stimulation paradigms, an even higher efficacy and tolerability of DBS is expected [18].

Interestingly, recent advancements in neurostimulator technology have enabled both the recording and streaming of local field potentials [20]which are a reflection of the coordinated electrical behaviour exhibited by a large population of neurons in close proximity to the electrode. However, DBS-electrodes cannot record single-neuron activity. Still DBS electrodes provide a unique window to investigate neuronal activity of targeted brain regions (currently sampling rate of 250 Hz with fully-implanted devices with streaming capacity [20]), record neuromarkers and enable DBS electrode-guided neurofeedback. While there are many similarities to EEG-neurofeedback, DBS electrode-guided neurofeedback does not rely on feedback from nebulous cortical areas but can intervene directly at the level of pathological circuits.

Herein, we will recount how Parkinson’s disease (PD) as the most common indication for DBS has been at the forefront of research using electrophysiological recordings from DBS electrodes. This research sparked the interest to investigate electrophysiological neurofeedback with implanted DBS electrodes. We will review the state-of-the-science with recent studies showing rapid learning of DBS electrode-guided neurofeedback in individuals with PD. We will also outline the most important challenges that need to be overcome for this technique to reach clinical application and be explored in other disorders. Furthermore, we explain how the large body of literature on non-invasive neurofeedback studies serves to inform us about potential novel DBS indications and motivates the use of DBS electrode-guided neurofeedback as an adjunct treatment modality to stimulation and medication.

### Motivating the use of DBS electrode-guided neurofeedback in parkinson’s disease

PD is the most common indication for DBS and has therefore paved the way towards DBS electrode-guided neurofeedback. PD is a highly prevalent and progressive neurodegenerative disorder and DBS can be considered as an effective treatment option for selected patients [21]. Typically, electrodes are placed either targeting the globus pallidus internus (GPi) or the subthalamic nucleus (STN), key motor relay structures for which dysfunction has been linked to PD symptoms [5]. While current evidence indicates that DBS can improve motor function in PD for over 10 years, some patients may not experience the desired level of symptom control [22, 23]. Moreover, some symptoms, such as postural instability, impaired balance, gait disturbances and difficulty swallowing, are known to be challenging to treat with DBS [22, 23]. In some patients, side effects limit the degree to which stimulation can be increased. Thus, the development of new or optimised therapies is one of the main actions aimed at addressing this great health challenge and improving the lives of those affected by PD.

Neurofeedback merits particular attention in the context of PD. The gradual loss of dopaminergic neurons in the basal ganglia and brainstem, fostering a dopamine deficiency resulting in major disruptions across basal ganglia-cortical networks, has sparked interest in exploring neurofeedback with real-time fMRI (rtfMRI) and more recently in a proof-of-concept fNIRS study [24]. With rtfMRI, the substantia nigra/ventral tegmental area complex (SN/VTA) could be voluntarily controlled through mental imagery in young, healthy participants [25]a region that is part of the pathological network in PD. Another group found that five patients with PD learned to increase activity in the supplementary motor complex and concomitantly influenced activity in basal ganglia circuits implicated in PD, resulting in significant and clinically relevant improvement of motor functions [26]. A follow-up randomised controlled trial (RCT) showed that patients in the neurofeedback group met the clinically important difference, whereas, however, the improvement did not differ significantly from the active control conditions. Larger trials are likely required to explore its superiority [27]. Nevertheless, rtfMRI is confined to experimental or clinical settings, expensive and complex to set up. While these studies demonstrated that participants can learn to gain control over deep brain areas, the methodological limitations could potentially be overcome with neurofeedback based on electrophysiological recordings.

Electrophysiological neurofeedback has been proposed given the growing body of evidence pointing towards the pathological role of abnormal neural oscillations in PD [28–30]. In particular, synchronised beta-oscillatory activity (13–30 Hz) seems to play a predominant role in the pathophysiology of PD. Beta-oscillations, which are thought to promote the current motor set (motor status quo) at the expense of new ones, are greatly enhanced in PD [31]and there is evidence for a link between beta-oscillations and Parkinsonian motor symptoms: Firstly, beta-activity recorded in the STN at rest in patients withdrawn from their medication has been correlated with the Unified Parkinson’s Disease Rating Scale (UPDRS) across patients [32, 33]. Secondly, a reduction of signal power in the beta-band was correlated with clinical improvements of motor symptoms [34–36]. Thirdly, the two main therapeutic strategies, high-frequency DBS [36, 37] and the administration of levodopa [38, 39]suppress beta-oscillations in the STN. Lastly, the movement-dependent modulation of beta-oscillations heavily depends on the behavioural context: externally-cued movements induce pro-kinetic beta-modulations while self-paced movements (which are often more affected in PD) do not [40, 41]. While the causal role of synchronised beta-oscillatory activity in Parkinsonian symptoms is still under investigation, there is evidence supporting this mechanistic link: stimulation to entrain cortical activity at 20 Hz in healthy subjects resulted in a slowing of voluntary movement [42]and imposed synchronisation through direct stimulation of the STN at a beta-frequency slowed motor performance in patients with PD [43]. It is thought that the beta-oscillations in PD may be an overexpression of normal cortico-basal ganglia-thalamic network dynamics due to a striatal dopamine/acetylcholine imbalance, rather than a de novo oscillation due to a PD-induced network pathology [44]. In summary, the observed electrophysiological changes are believed to be pathological manifestations of PD and are closely linked to its symptoms. This understanding opens avenues for causal interventions, such as electrophysiological neurofeedback, aimed at modulating these changes. Such interventions can be instrumental in assessing their impact on symptoms and may serve as valuable adjuncts to existing treatments.

To date, a number of studies have assessed the efficacy of electrophysiological neurofeedback with scalp EEG in PD [29]even demonstrating its feasibility in a home setting [45]. These studies provide initial evidence that patients with PD can modulate cortical activity at multiple sites with EEG-neurofeedback, but the impact on behavioural and clinical metrics is, as of now, less clear. However, EEG-neurofeedback is cumbersome to set up and signal quality as well as spatial resolution are an issue. Compared to EEG-neurofeedback, invasive electrocorticography provides a spatially more focal approach with a higher signal-to-noise ratio. Using this modality, three PD patients were successful at controlling cortical beta-band power from sensorimotor areas through electrical neurofeedback [46]. Since DBS has demonstrated its safety and efficacy in the treatment of PD, prompting its consideration earlier in the disease course [47]and with the latest generation of DBS systems capable of recording and streaming LFPs around implanted electrodes [48]there is now ample opportunity to investigate the potential of fully-implanted devices for DBS electrode-guided self-modulation of target locations. This approach thus provides unique access to investigate neurofeedback-modulation of neural oscillations at a DBS target location.

### State-of-the-science of DBS electrode-guided neurofeedback in parkinson’s disease

The wide clinical use of therapeutic DBS of the STN or GPi provides an unprecedented window into basal ganglia neurophysiology that can be leveraged to investigate DBS electrode-guided neurofeedback. First studies explored DBS electrode-guided neurofeedback using temporarily externalised DBS leads, which were connected to an external amplifier and recording device: Measurements occurred either during surgery, with local anaesthesia for replacement of the implantable pulse generator [49]or after the first surgery for electrode implantation and prior to the second operation to connect the electrode(s) to the implantable pulse generator [50–52].

The study performed during the replacement of the implantable pulse generators was conducted with eight PD patients ON dopaminergic medication [49, 53]. Over a period of 10 min, they were trained to either upregulate (4 patients) or downregulate (4 patients) subthalamic beta-power. The 5 min average resting subthalamic beta-power was evaluated as an outcome and all four patients in the downregulation group showed a significantly reduced beta-power post-feedback as compared to pre-feedback. Yet, only two out of four patients in the upregulation cohort showed significantly increased values, possibly related to ceiling effects in the beta-signal despite regular dopaminergic medication [28]. Nevertheless, it remained to be unveiled whether DBS electrode-guided neurofeedback allows for *instantaneous* modulation, whether individuals can achieve bidirectional control, and whether modulatory effects improve with exposure time and persist after removal of neurofeedback. We demonstrated the ability to modulate STN beta-power in 8 patients with externalised DBS leads [50]. Within as little as a single 60 min training session (and only 6 min of effective downregulation training), the reduction of beta-oscillations progressively improved to a median reduction of 15% in the final neurofeedback block. Neurofeedback-learnt strategies were retained in the short-term, as suggested by the endogenous control over STN beta-activity even in the absence of ongoing neurofeedback. A study from another group investigated three patients with PD targeting beta bursts, rather than conventional beta power, among whom two patients achieved significant control over the appearance of subthalamic beta-bursts [51]. Lastly, twelve patients with PD showed reduced beta-synchrony as well as reduced beta-band coupling between the STN and motor cortex after neurofeedback training [52]thereby hinting at a lasting effect following active neurofeedback. While these studies using externalised DBS leads support beta-oscillations as a target for DBS electrode-guided neurofeedback, externalised leads are neither suitable for every-day neurofeedback-use nor larger and long-term studies.

In order to transfer the applicability of DBS electrode-guided neurofeedback to the every-day setting, we reproduced these promising results of beta-power regulation with, for the first time, a fully implanted DBS system [54]. While receiving continuous deep brain stimulation and dopaminergic medication, 8 participants with PD could significantly improve their neurofeedback ability and achieved a significant decrease of subthalamic beta-power (median reduction of 31% in the final neurofeedback block, after only 4 min of prior downregulation learning). When pooling the results from our two studies [50, 54]this so far largest single-centre cohort with DBS electrode-guided neurofeedback demonstrated the successful downregulation of pathological oscillations in all 16 participating patients. The results of the two studies are highly comparable, despite different signal processing (full wave rectification and *n*-point moving average in the externalised DBS leads vs. fast Fourier transform with the fully-implantable solution [20]) and visual representation of the neurofeedback parameter (horizontal movement of a disk from lower values on the left to higher values on the right at an update rate of 25 Hz vs. running average graph updated at 2 Hz). Furthermore, the results support the generalisability of our findings due to the following differing aspects between the two studies: mix of DBS leads (Model 3389 vs. SenSight™ of Medtronic), different sampling rate (5’000 Hz vs. 250 Hz), varying amount of days after surgery (3–5 vs. 3–30 days after electrode implantation) as well as DBS OFF vs. DBS ON. Especially for the clinically more relevant downregulation task (as compared to the also investigated upregulation task), we achieved consistent results in the two studies using the same protocol for the neurofeedback experiments. The bidirectional design of the neurofeedback experiment permitted an intra-individual control such that differences in beta-power would be solely explained by the neurofeedback condition. The amount of beta-power reduction is considerable, as demonstrated by the last neurofeedback round of our second study, where patients achieved a mean decrease of ~ 30% for downregulation vs. rest. For comparison, other studies with levodopa described a beta-power decrease of 54–59% and 45.8% for a DBS-only therapy [54]. This is remarkable, as the neurofeedback results were obtained under concurrent DBS and pharmacological therapy (note that, as the study was performed during the rehabilitation phase, DBS was increased slowly over days and was most frequently not in the optimal setting). In summary, the replication of the promising beta-modulatory capacity through externalised DBS leads in a fully-implantable solution offering DBS electrode-guided neurofeedback motivates the broader investigation of this technique in large-scale, long-term trials.

While previous studies demonstrated the suppression of pathological beta-oscillations after DBS electrode-guided neurofeedback, the effect of this intervention on motor symptoms is less clear as of now. In our first study [50]we captured the motor effects of neurofeedback during the last neurofeedback round, where each block was immediately followed by 15 s of pro- and supination movements of the symptom-dominant hand, performed as fast and complete (i.e. 180°) as possible. We observed improved motor control during a single one-hour training session: The movement frequency was higher (median increase of 9%) and the absolute angular acceleration as a proxy for the antagonistically acting pro- and supinator muscles was greater (median increase of 11%) directly after neurofeedback-guided beta-downregulation. The study which showed a reduced beta-synchrony within the STN as well as reduced beta-band coupling between the motor cortex and the STN after neurofeedback training in twelve patients also investigated the effects of this intervention on motor symptoms [52]. They found reduced reaction times in subsequently cued movements. However, in Parkinsonian patients with pre-existing symptoms of tremor, successful volitional beta-suppression was associated with an amplification of tremor which correlated with theta-band activity in STN local field potentials, suggesting an additional cross-frequency interaction between STN beta- and theta-activities. In a nutshell, stronger evidence for the link of DBS electrode-guided beta-neurofeedback with improved motor outcomes yet needs to be provided, with some studies as of now demonstrating improved motor outcomes, some finding mixed results or no significant relationship, and many not assessing any behavioural or clinical metrics at all.

### Towards chronic DBS electrode-guided neurofeedback in parkinson’s disease

**Fig. 1:**
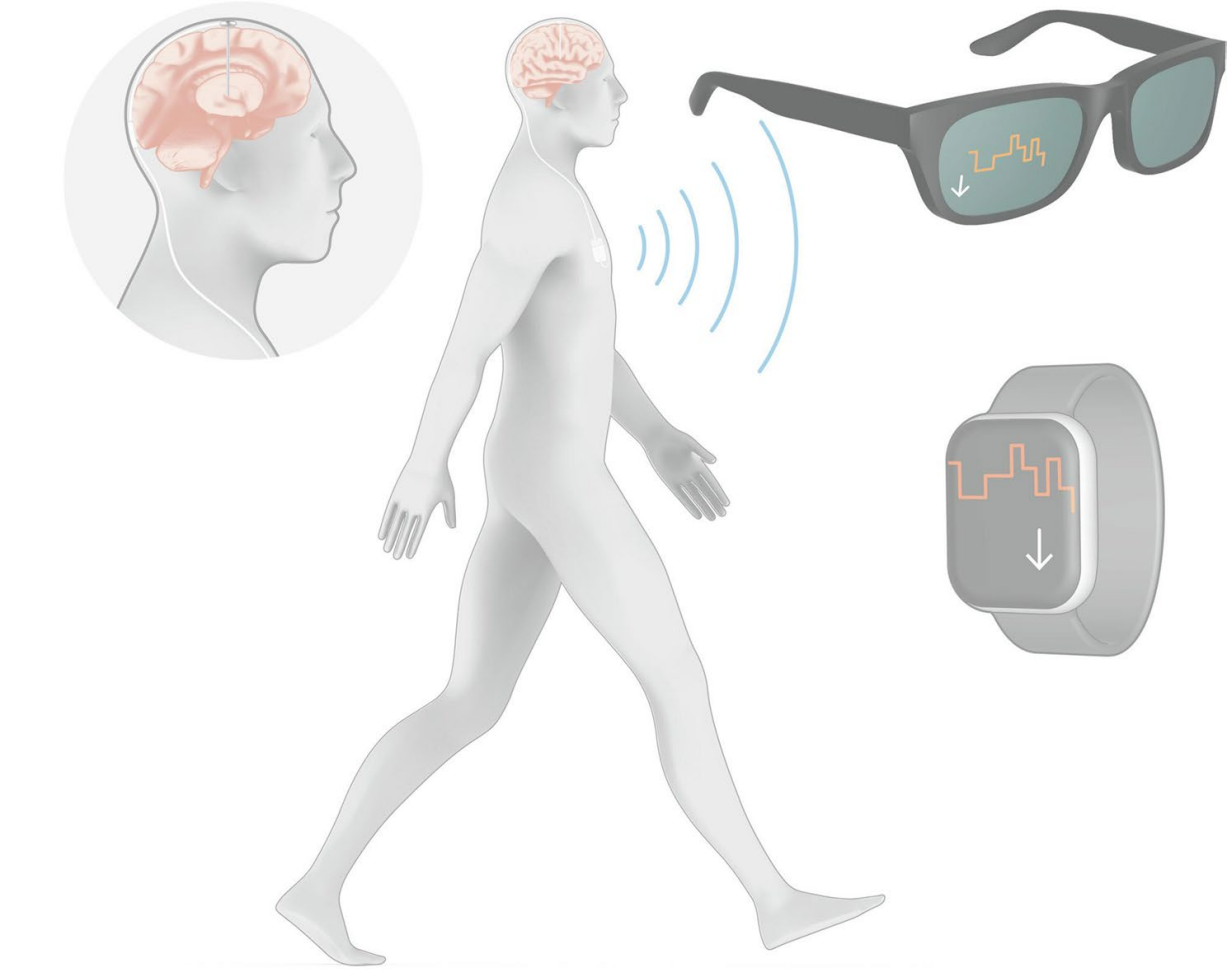
A vision for DBS electrode-guided neurofeedback with simultaneous stimulation and streaming to a smart watch and/or augmented reality glasses. The DBS system is fully implanted and can be used in every-day settings to enable DBS electrode-guided neurofeedback, thereby incorporating patients into the treatment loop. In this case, visual neurofeedback in the form of a stepped time-course is provided to a smart watch and/or augmented reality glasses with an arrow indicating the target direction. So far, visualisation has been provided on a computer screen or tablet

There is evidence that DBS electrode-guided neurofeedback in Parkinson’s disease over longer time-periods could potentiate its effectiveness. In most EEG-neurofeedback studies, training sessions are repeated over separate days [55, 56]. Along this line, improved beta-control with DBS electrode-guided neurofeedback has been shown to improve over several rounds in a single day [50] as well as over-night [52]. Moreover, in our two studies, PD patients, after only 6 min of effective downregulation training and in the absence of ongoing neurofeedback, were still able to significantly reduce subthalamic beta-power with their previously acquired neurofeedback strategies [50, 54]. Nevertheless, downregulation without visual neurofeedback was less effective than with active visual neurofeedback, further motivating the long-term or chronic use of DBS electrode-guided neurofeedback. With certain novel neurostimulators having the capacity to record (interleaved with stimulation) and wirelessly stream LFPs, DBS electrode-guided neurofeedback no longer relies on externalised DBS electrodes. We provided first evidence that neurofeedback training with a fully-implanted system for DBS with streaming ability was successful [54]thus presenting proof-of-principle of a system for DBS electrode-guided neurofeedback that could be employed in every-day life [57] (Fig. 1).

We reviewed the evidence of DBS electrode-guided neurofeedback enabling the self-control over pathological deep brain oscillations in PD, which in turn led to an improved motor performance in some cohorts. As PD symptoms strongly fluctuate, neurofeedback-acquired strategies to reduce pathological subthalamic oscillations could assist patients in overcoming transient exacerbations, complementing DBS and medication by a fast-acting *endogenous* approach. This could augment the therapeutic armamentarium by a near-instantaneous, nuanced neurofeedback approach contrasting the tardiness of medications and tonic effect of constant DBS paradigms. Moreover, neurofeedback-acquired strategies might have less adverse side-effects on functional circuits than *exogenous* therapies, such as for instance those of dopamine or stimulation. Thus, DBS electrode-guided neurofeedback could potentially be employed in situations where a higher medication or stimulation dose would result in (pronounced) side-effects. Moreover, DBS electrode-guided neurofeedback might also reduce the overall exposure to brain stimulation and medication, thereby antagonising the development of tolerance. Ultimately, patients with PD could be reincorporated into the treatment loop through DBS electrode-guided neurofeedback: Whereas PD patients are mere bystanders with only little control over their burden and *exogenous* therapy, neurofeedback-acquired control over pathological oscillations could be a means to increase agency by actively and *endogenously* reducing medication as well as stimulation load while simultaneously alleviating symptoms. It yet remains to be unveiled whether *endogenous* neuromodulation of deep brain circuits leads to more beneficial long-term neuroplastic changes as compared to the ones achieved through DBS [58]; The *exogenous* application of DBS might be less-nuanced and less physiological. As previously mentioned, some symptoms, such as postural instability, gait and other axial symptoms are known to be challenging to treat with traditional DBS. This could be attributed to the possibility that these particular symptoms may not be effectively targeted by the current DBS circuit or nodal points. In considering alternatives for these DBS resistant disease features, a thorough investigation of DBS electrode-guided neurofeedback might offer more selective and physiological (endogenous) approaches to modulating the underlying neural mechanisms once relevant biomarkers have been identified for these DBS-resistant symptoms. While so far not the primary focus of neurofeedback studies in PD, non-motor symptoms could also be targeted by DBS electrode-guided neurofeedback and there are first promising findings from EEG and fMRI studies [30]. Moreover, in cases where DBS is limited by side-effects, which in many cases could relate to off-target electrode placement, DBS electrode-guided neurofeedback approaches might remain efficacious when symptom-specific biomarkers can still be reliably picked up despite a reduced signal-to-noise ratio. In short, DBS electrode-guided neurofeedback could be a powerful strategy for patients with PD and thus potentially complement current treatment modalities.

### DBS electrode-guided neurofeedback for other neurological and psychiatric disorders

With DBS-electrode guided neurofeedback recently emerging as a potential adjunct treatment to stimulation and medication in patients with PD, its exploration in a wider range of neurological and psychiatric indications is motivated. The most likely next candidate indications are disorders where DBS is already established or being explored, such as essential tremor, dystonia, obsessive-compulsive disorder, epilepsy, major depressive disorder and Tourette syndrome. Moreover, the growing body of neurofeedback research might identify novel targets to test DBS electrode-guided neurofeedback in further neurological and psychiatric indications [6]. Additionally, and importantly, this technique can also be a powerful tool to study the human brain, helping to unravel important details of the pathophysiology of underlying disorders of circuit functions. Potential DBS target locations are numerous and encompassed by, inter alia, motor, sensory, interoceptive, limbic, cognitive, mood, reward, motivation and memory circuits. Within these circuits, volitional regulation through DBS electrode-guided neurofeedback allows causal inferences to be made between brain activity and behaviour as well as patient reports. DBS electrode-guided neurofeedback has the potential to interrogate brain circuits with a higher signal-to-noise ratio and temporal resolution (as compared to fMRI), which in combination with its advantage to record signal over extended time-spans could identify promising biomarkers. Once neuronal markers for symptoms are better characterised, this information can later also be used to fine-tune DBS parameters for optimal stimulation. DBS electrode-guided neurofeedback could also be combined with, e.g., fNIRS or EEG in a multi-modal neurofeedback design, thus interrogating cortico-subcortical circuits with probes at both levels.

The generalisation of the effect of DBS electrode-guided neurofeedback is yet pending the proof of concept in other indications besides PD. Most importantly, the feasibility of DBS electrode-guided neurofeedback depends on the identification of a robust, disease-specific neurophysiological biomarker. Therefore, in future applications, a combination of biomarker-supported neurostimulation and biomarker-controlled neurofeedback could be aimed at and possibly achieve synergistic effects. One distinct advantage of DBS electrode-guided neurofeedback is that it may provide a means for modulating oscillations in different frequencies with more selectivity as opposed to the less electrophysiological changes achieved by DBS [58] or medication [59]. Moreover, endogenously controlling activity may over time promote self-regulation or even neural plasticity [60].

### DBS electrode-guided neurofeedback for pain

Pain serves a vital function as an unpleasant sensory and emotional experience associated with, or resembling that associated with, actual or potential tissue damage [61]. Unlike acute pain, chronic pain syndromes linger persistently without a clear protective benefit, thereby posing a major healthcare problem and constituting leading contributors to disability worldwide [62]. Chronic pain demands specialised attention and targeted interventions for effective management. Conventional therapeutic modalities such as behavioural therapy and pharmacotherapy, while beneficial for many, often fall short in adequately controlling symptoms in a subset of patients, leading to the exploration of innovative neuromodulatory approaches [48, 63]. Non-invasive neurofeedback has already been shown to be a safe and effective therapy with promising evidence supporting its use in chronic pain [64–66]. It could offer a non-addictive, non-pharmacological approach to pain management, thereby also allowing to better cope with possible side-effects of pharmacotherapy. Yet, in our opinion, it seems unlikely that these non-invasive neurofeedback approaches can be effective enough in controlling severe forms of chronic pain (refractory to behavioural therapy and pharmacotherapy), which are the same cases in which DBS has emerged as a potential treatment option [48]. In a first step, DBS electrode-guided neurofeedback could be used as an adjunct to ongoing stimulation, which would then also justify the surgery with its associated risks.

Central or peripheral neurological insults can lead to long-lasting pathological changes in various brain regions involved in pain processing that can be broadly categorised into regions that process somatosensory, affective and cognitive information [67]. Spontaneous pain can be generated through spontaneous ectopic discharges (due to a direct lesion or secondary to deafferentation) within central nociceptive pathways [68–70]. Moreover, by direct electrical stimulation of the posterior insula in humans, pain during stimulation could be elicited, supporting that pain can be generated by activity in these brain areas [71]. DBS targets within pain networks have been investigated for a plethora of pain indications and included the periaqueductal grey and periventricular grey matter region, the ventral thalamus, the anterior cingulate cortex, the ventral striatum and anterior limb of the internal capsule [72]. The mixed results obtained in these clinical studies showing only limited efficacy over time with DBS is likely due to the current one-size-fits-all approach. Optimal outcomes necessitate a personalised and patient-specific approach, considering factors such as the type, location, and severity of pain, as well as individuals’ response to stimulation. With the advent of advanced neuroimaging techniques, such as fMRI and DTI-MR-tractography [18]it is nowadays possible to reveal areas active in pain processing and study their connectivity, respectively, to optimise DBS target selection on an individual level.

Neuronal biomarkers for pain have been proposed for chronic pain syndromes and include theta, alpha and gamma oscillations in the primary somatosensory cortex, the anterior cingulate cortex, and the orbitofrontal cortex [67]. Recently, a study demonstrated that intracranial orbitofrontal cortex signals could be used to predict spontaneous, chronic pain states in four individuals [73]. Such promising neuronal biomarkers offer the potential to enhance DBS electrode-guided neurofeedback, aiming for personalised interventions that could lead to better outcomes in patients with treatment-resistant chronic pain syndromes.

### DBS-electrode guided neurofeedback for epilepsy

In recent years, a paradigm shift has taken place in epilepsy research towards an understanding of epilepsy as a network disease. While seizure onset is mainly in cortical areas, ictal propagation, seizure spread and seizure termination are mediated through comprehensive brain networks including deep brain structures, such as the thalamus or basal ganglia. In the recent operative definition of epilepsy, the International League Against Epilepsy [74] therefore propagates the view of epilepsy as a network dysfunction mediated through a mutual interaction of cortical and subcortical brain structures. This renewed disease concept also provides a basis for novel treatment options targeting the pathological epileptic network. Current standard treatment for epilepsy are pharmacotherapy (whole brain approach) or resection of the seizure focus (invasive focal approach) to disrupt seizure onset. However, by targeting brain *networks*, neurostimulation techniques are a novel promising treatment option for patients with drug-resistant epilepsy who are refractory to medication and not considered candidates for focal resective epilepsy surgery due to factors such as a bilateral, multifocal or generalised seizure onset or seizure origin within highly eloquent cortex. From a clinical perspective, the landmark study by Fisher et al. has provided comprehensive evidence that the epileptic network can indeed be modulated in a therapeutic way by high frequency stimulation in the thalamus in the acute and chronic phase [75, 76]. The most common targets for DBS in epilepsy include the anterior nucleus of the thalamus, the centro-median nucleus of the thalamus and the hippocampus, as these regions are commonly involved in the generation and propagation of seizures. While the neurophysiological mechanisms for the efficacy of thalamic DBS in epilepsy are currently being investigated, it is believed to modulate abnormal neuronal activity and disrupt the propagation of signals within the epileptic network. In line with this, it has recently been shown that, similar to PD, frequency-specific biomarkers of the LFP can be identified in deep brain structures. In contrast to PD, these oscillations are found in the thalamus and several studies highlight the correlation of theta-band activity with increased epileptic activity before, during and after seizures. For instance, functional connectivity between the thalamus and the hippocampus is increased during seizures (as shown in an invasive study in patients during presurgical evaluation) [77]. Furthermore, high frequency stimulation of the posterior thalamus leads to seizure termination of hippocampal seizures [78].

Recently, theta band activity in the anterior thalamus was investigated in the inter-ictal phase in a cohort of patients undergoing implantation of deep brain electrodes for chronic ANT-DBS [79]. This study also revealed higher theta band activity in patients who later were responders to ANT-DBS treatment. Theta band activity in the thalamus might therefore be a suitable, comprehensive biomarker for the pathological epileptic network state. Prospective studies targeting this biomarker for prognosis, DBS treatment adaptation or biomarker-guided closed loop stimulation are still missing. However, these striking parallels between PD and epilepsy suggest that this novel biomarker might prove useful for DBS electrode-guided neurofeedback also in epilepsy patients. Given that thalamic theta-activity is increased during seizures, can be reduced by DBS and shows a fluctuating temporal variability within and between patients, DBS electrode-guided neurofeedback might be an additional option for reducing the pathological network activity in epilepsy and could eventually lead to an improvement in disease symptoms, particularly seizure frequency and severity.

### DBS electrode-guided neurofeedback for stroke

Stroke is a leading cause of disability worldwide [1] with survivors experiencing significant disability and reduced quality of life related to ongoing maladaptive responses (e.g. central neuropathic pain, movement disorders, epilepsy) and persistent neurological deficits. For many patients, both the persistent deficits and the maladaptive responses prove refractory to conventional therapy and have prompted the investigation of various forms of brain stimulation, including DBS especially for maladaptive phenomena [80]. The further approach with DBS electrode-guided neurofeedback would likely follow the framework for its application in epilepsy and pain as delineated above. Established DBS electrode-guided neurofeedback strategies for certain movement disorders could be adopted as an adjunct treatment modality to the management of cerebral palsy [81–83] or stroke-related movement disorders. Yet, aetiology-specific differences in plasticity, disease course and underlying brain networks should be carefully considered.

The evidence supporting the utility of DBS in improving post-stroke deficits is, however, as of now less compelling [80]. Cerebellar DBS has been clinically expored [84] and a clinical trial of cerebellar DBS for post-stroke rehabilitation was recently initiated after positive preclinical results had been obtained [85]. As of now, it is unclear whether DBS is able to produce significant results on its own. Optimal results will likely require multifaceted approaches combining DBS with other, complementary neuromodulation techniques. Neurofeedback has shown recent success in initial RCTs for the use of this application in rehabilitation after stroke [6]. For instance, promising evidence of neurofeedback efficacy after stroke has come from a sham-controlled, double-blinded RCT in severely impaired, chronic patients that took a novel neurofeedback–brain-machine-interface approach with adjuvant physiotherapy. These patients learned to upregulate ipsilesional sensorimotor areas by reinforcing successful mu-rhythm desynchronisation with robotically assisted hand manipulation [86]. A similar approach using EEG-neurofeedback with action-observation therapy in a subacute-stroke RCT found a remarkable (clinically meaningful) 8.1 point improvement in upper-limb Fugl-Meyer score relative to controls who used only mental imagery [87]. Building on this, thalamic DBS could be envisioned to upregulate ipsilesional sensorimotor areas and multi-modal neurofeedback through DBS electrode-guided neurofeedback (pending the identification of suitable biomarkers) with EEG or fNIRS used to target specific cortico-basal ganglia-thalamic circuits. It then remains to be investigated whether such a neurofeedback-based approach could outperform stimulation alone regarding behavioural metrics, given its more physiological network activation.

### Limitations, open challenges and recommendations

DBS electrode-guided neurofeedback is a promising technique and first studies have shown that PD patients can rapidly gain control over features which have been associated with disease severity. Some of these have demonstrated improved motor outcomes, some found mixed results or no significant relationship with behaviour, and many did not investigate behavioural or clinical metrics at all. For future studies, researchers should adhere to the consensus on the reporting and experimental design of clinical and cognitive-behavioural neurofeedback studies (CRED-nf checklist) [88] in order to maximise the value of their efforts. Of special note are the items pertaining to pre-registration of study, control groups and measures, as well as outcome measures [29, 30]. In terms of preregistration, authors should consider submitting a registered report, where initial peer-review and in-principle acceptance for publication occur before knowing the research outcomes. Recent evidence suggests that registered reports could improve research quality while reducing publication bias [89–91]. Furthermore, there is a potential role for international consortia, such as the ENIGMA Neuromodulation Working Group [92]to provide a community-driven structure and platform for collaborations to assist the planning and conduction of well-powered, multi-centre studies. There are also technological challenges that need to be overcome. The draining effect of neurofeedback on the implanted battery can probably be neglected with rechargeable devices (patients recharge their devices every X days, depending on usage patterns and battery life), when streaming is optimised or DBS electrode-guided neurofeedback can reduce stimulation dose. Yet, it is important not to neglect the risk for infection as well as other challenges that more frequent battery replacement surgeries in non-rechargeable devices could bring in situations where optimised streaming and neurofeedback-related reduction of stimulation dose still result in increased energy consumption compared to treatment without neurofeedback. As neurostimulators equipped with streaming capability are increasingly employed more frequently and some are even rechargeable, more patients could agree to participate in DBS electrode-guided neurofeedback studies. Besides, paralleling the efforts in neurofeedback research, the community should also perform studies over extended periods in order to explore if there is a learning effect over time, which could allow to modulate pathological signals even more strongly and thus result in larger behavioural benefits. For efficient neurofeedback, requirements for sampling rate and optimised signal quality also need to be unveiled. Neurofeedback presentations should also be made more efficient as well as engaging; So far, they only consisted of rather simple visual feedback and did not show the evolution of success. As of now, the restricted permission to adapt visual feedback due to producers’ limitations is a factor that needs to be urgently tackled in close collaboration with industrial partners. Moreover, the innovation in this field is limited since currently only one large provider for DBS pulse generators provides devices with streaming capacity.

Patient adoption of DBS electrode-guided neurofeedback is an anticipated challenge should there be no relevant carry-over benefit. This potential challenge underscores the importance of balancing perceived benefits with the burdens imposed on patients. While the potential for symptom alleviation or disease management through DBS electrode-guided neurofeedback interventions is promising, the requirement for sustained focus and attention from patients poses a significant barrier to adoption. To overcome this, future research should also focus on less obtrusive neurofeedback modalities, such as haptic neurofeedback, as well as DBS electrode-guided neurofeedback in parallel to ongoing DBS. Additionally, early clinical trials of closed-loop DBS systems (featuring a novel function where stimulation parameters are continuously adapted based on a neural biomarker) [93] prompt us to consider a supportive role of DBS electrode-guided neurofeedback. Given that neurofeedback can target abnormal oscillations, and thereby potentially also the biomarker controlling the adaptation of DBS settings, we hypothesise that patients with closed-loop DBS systems might require less stimulation while performing neurofeedback whilst their clinical benefits are preserved. Ultimately, DBS electrode-guided neurofeedback relies on a clinically indicated neurosurgical procedure and requires a thorough risk-benefit analysis.

## Conclusion

We herein discussed the transition from first results of DBS electrode-guided neurofeedback with externalised electrodes that could be replicated with a fully implantable solution using state-of-the-science neuromodulators with streaming capacity. As current studies were limited by short investigational windows, the full extent to which voluntary self-modulation of deep brain oscillations is possible and improves behavioural outcome yet remains to be unravelled. Nevertheless, neurofeedback based on both non-invasive and invasive imaging is gaining strong momentum in the research community, with promising results in various clinical populations. We provide an outlook for other indications where DBS is currently used or explored and indications where neurofeedback has been shown beneficial with non-invasive modalities, as we believe that DBS electrode-guided neurofeedback can potentially be generalised and employed in other brain circuit dysfunctions. Yet, despite its promise for the treatment of various neurological and psychiatric disorders, it is not without its shortcomings and challenges. Technological advancements and continued research adhering to community guidelines are required to overcome these hurdles and improve patient outcomes.

## Data Availability

No datasets were generated or analysed during the current study.
